# Dexamethasone implants in paediatric patients with noninfectious intermediate or posterior uveitis: first prospective exploratory case series

**DOI:** 10.1186/s12886-017-0648-3

**Published:** 2017-12-16

**Authors:** Sibylle Winterhalter, Uwe Diedrich Behrens, Daniel Salchow, Antonia M. Joussen, Uwe Pleyer

**Affiliations:** 1 Department of Ophthalmology, Campus Virchow- Klinikum, Charité – University Medicine Berlin, corporate member of Freie Universität Berlin, Humboldt- Universität zu Berlin and Berlin Institute of Health, Berlin, Germany; 2 Coordination Center for Clinical Studies, Campus Virchow- Klinikum, Charité – University Medicine Berlin, corporate member of Freie Universität Berlin, Humboldt- Universität zu Berlin and Berlin Institute of Health, Berlin, Germany

**Keywords:** Paediatric patients, Uveitis, Dexamethasone implant, Glaucoma, Cataract, Vitreous haze, Macular edema

## Abstract

**Background:**

To evaluate the efficacy and safety of dexamethasone (DEX) implants in paediatric patients with noninfectious intermediate or posterior uveitis.

**Methods:**

Prospective single center exploratory case series. Children and adolescents, 6 to 17 years old, with a vitreous haze score of ≥1.5+ or cystoid macular edema (CME) of >300 μm were enrolled. Vitreous haze score at month 2 was chosen as primary endpoint. Best corrected visual acuity (BCVA), central retinal thickness (CRT) and concomitant medication at month 6 were defined as secondary endpoints. Intraocular pressure (IOP) and cataract formation were determined as safety endpoints.

**Results:**

Three out of 6 eligible patients participated in the case series. At month 2, vitreous haze was reduced from a score of 1.5+ to 0.5+ and 0 and BCVA improved by ≥3 lines, ≥4 lines and ≥2 lines of Early Treatment of Diabetic Retinopathy (ETDRS)-letters, respectively. Visual acuity gain was accompanied by a CRT reduction of −186 μm and −83 μm in the first and third patient, in whom CME was the indication for DEX implantation. A reduction of concomitant medication was achieved in 1 patient. IOP increase was seen in all 3 patients, but could be treated sufficiently with primarily IOP lowering medications and without need for glaucoma surgery. Cataract progression did not occur.

**Conclusions:**

DEX implants led to an improvement in all endpoints, especially BCVA. This study confirms that IOP rises may also occur in the paediatric population and should be monitored and treated appropriately.

**Trial registration:**

European Union Drug Regulating Authorities Clinical Trials (EudraCT)- nr: 2013–000541-39.

## Background

Uveitis in childhood has an incidence of 4.3–4.9/100.000 [1, 2] and accounts for 2–9% of all uveitis patients. Of these children, 10–12% have intermediate uveitis and may experience vision-threatening complications such as cataract formation, macular edema, glaucoma and amblyopia [3, 4].

In intermediate and posterior uveitis topical steroids are usually not effective enough and systemic treatment may be necessary. Systemic steroids can lead to fast control of inflammation but are frequently accompanied by systemic side effects including cushingoid habitus, weight gain, increased blood pressure, gastrointestinal discomfort and ulcera, steroid induced diabetes and psychosis, insomnia, osteoporosis, electrolyte disbalance and growth retardation. Ocular side effects include cataract formation and increased intraocular pressure (IOP). Therefore, steroid-sparing agents are often applied earlier in children and adolescents than in adults [5, 6]. Methotrexate (MTX) is often the treatment of first choice in paediatric patients with intermediate or posterior uveitis, particularly if associated with juvenile idiopathic arthritis (JIA) [6–8]. One disadvantage of MTX is its delayed therapeutic effect that may take up to 6–10 weeks to set in. Possible side effects of MTX include gastrointestinal and hematologic toxicity, increase of liver enzymes and cirrhosis, pneumonitis, pulmonary fibrosis and teratogenicity. If MTX is not effective enough, Azathioprine, Ciclosporine A (CSA) or biologicals (Adalimumab, Infliximab) may be used [9–11].

Unfortunately, uveitis is not always controlled, even with an intensified local and systemic treatment [12]. In such children and adolescents, an intravitreal steroid application may be effective. Dexamethasone (DEX) implants have been approved by the European Medicines Agency (EMA) [13] for the treatment of non-infectious uveitis of the posterior segment in adults and may be effective for up to 6 months. Systemic side effects appear to be absent, but the treatment has not been approved in paediatric patients.

Adverse effects of intravitreally applied steroids include cataract formation and increase of IOP [14, 15]. So far, reported numbers of patients with these adverse effects have been low in all published studies [13, 16–27]. To date, safety and efficacy of DEX implants in paediatric patients have not been established. The aim of our case series was to prospectively investigate the efficacy and safety of DEX inserts in children and adolescents with intermediate and posterior uveitis.

## Methods

This prospective single center case series was planned in an exploratory layout. Approval of the German Institute for Medicinal Products and Medical Devices (BfArM) and the Institutional Review Board (IRB) of the Charité-Universitätsmedizin Berlin (13/0282 – EK 15) were obtained. The case series was registered under the European Union Drug Regulating Autorities Clinical Trials (EudraCT) number 2013–000541-39 and was conducted in concordance with the good clinical practice (GCP) regulations and the German Law for Medicinal Products (AMG).

### Main inclusion criteria

Patients, aged 6–17 years, with non infectious intermediate or posterior uveitis were included. Additional inclusion criteria were:

- Vitreous haze (VH) ≥1.5+ on a scale from 0 to 4 or cystoid macular edema (CME) with central retinal thickness (CRT) >300 μm
- Best corrected visual acuity (BCVA) of 10 to 75 Early Treatment of Diabetic Retinopathy (ETDRS) letters (corresponding to 20/630 to 20/32 on the Snellen chart) [28]
- Permitted medication, if stable for at least 2 weeks:
  - topical corticosteroids
  - systemic immunosuppression
  - systemic corticosteroids <20 mg/d

If both eyes were eligible for enrollment, the worse eye was chosen for this study.

### Main exclusion criteria

Patients were excluded if active ocular infection was present in the study eye, if they had hypersensitivity to dexamethasone or any other components of the DEX implant and if aphakia was present. Eyes with advanced glaucoma or with insufficient IOP control and eyes with a known steroid response were not enrolled. Furthermore, patients with previous treatment with DEX- or Fluocinolone acetonide (FA)- implants were excluded, as were patients who had received periocular corticosteroid injections within 12 weeks or intravitreally applied steroids within 26 weeks prior to screening.

### Examinations and follow- up

The screening visit and follow up visits included:

1. BCVA according to the ETDRS criteria [28]
2. CRT measured by Spectral Domain Optical Coherence Tomography (SD-OCT) (Heidelberg Eye Explorer Version 6.011.0, Heidelberg Spectralis, Heidelberg Engineering, Heidelberg, Germany)
3. IOP measurement
4. Slitlamp examination of the anterior segment and anterior vitreous
5. Grading of any lens opacities according to the “lens opacity classification system” (LOCS)
6. Funduscopy including assessment of vitreous haze (VH) according to the Nussenblatt scales [29]
7. History of medication use
8. Assesment of in- and exclusion criteria
9. Written informed consent of the parents to participate in this case series

Follow-up examinations were performed at day 1 and 7 after DEX injection. Further follow-up visits were scheduled monthly until month 6 after DEX implantation. Adverse and serious adverse events (AE, SAE) were recorded at every study visit.

### Treatment

DEX injections were performed under sterile surgical conditions (operating theatre, topical anaesthesia in adolescents and general anaesthesia in younger children, using a tunnel technique via the pars plana) by an experienced uveitis and retina specialist (SW).

### Primary and secondary study endpoints

Primary study endpoint was VH score at month 2 after DEX injection. BCVA, CRT and use of concomitant medications were defined as secondary endpoints at month 6. Safety endpoints were IOP and cataract formation.

### Withdrawal, rescue and re- treatment criteria

Study withdrawal criteria were uncontrolled glaucoma and presence of endophthalmitis. Rescue treatment with oral steroids could be applied if vitreous haze score increased by ≥1+ unit from week 4 to week 8 or by ≥1.5+ units from week 8 to week 26. A re-treatment with DEX implant could be performed if the VH score increased by ≥1+ unit after initial reduction or if CME worsened (defined as an increase of central retinal thickness to >300 μm after prior reduction), or if a loss of at least 10 letters in BCVA occurred due to active inflammation.

## Results

Between March 2014 and June 2015, six patients were identified as eligible for the study. Three patients met the inclusion and exclusion criteria (Table 1) and were included into the study after written informed consent of the parents was obtained. The remaining patients did not meet the study criteria because of known IOP increase in response to steroid therapy or because they refused to participate. Table 2 shows the results of primary and secondary endpoints and safety endpoints. The additional Figs. 1, 2 and 3 show in more detail BCVA, CRT and IOP during study course.

**Table 1:** Patients demographics at baseline

	Patient 1	Patient 2	Patient 3
Gender	female	male	male
Age in years	17	9	17
Localisation of uveitis	intermediate	intermediate	intermediate
Study eye	LE	LE	RE
Indication, symptoms	CME	VH	CME, VH
Underlying disease	no	Suspicion of sarcoidosis	no
Medication	CSA 2.1 mg/kg body weight daily	Decortin 0.2 mg/kg body weight once daily	MTX 7.5 mg/m^2^/once weekly; Prednisolone 0.03 mg/kg body weight once daily
BCVA study eye (ETDRS-letters)	65	55	58
IOP study eye	18	17	18
CDR study eye	0.2	0.2	0.1
Lens status study eye	clear	clear	Subcapsular cataract grade 2+
CRT study eye (μm)	451	320	350
VH study eye	0.5+	1.5+	1.5+
VC study eye	0.5+	0.5+	1+

*RA* right eye, *LE* left eye, *CME* cystoid macular edema, *VH* vitreous haze, *CSA* Ciclosporine A, *MTX* Methotrexate, *ETDRS- letters* Early Treatment of Diabetic Retinopathy- letters, *IOP* intraocular pressure, *CDR* cup/ disc- ratio optic nerve head, *CRT* central retinal thickness, *VC* vitreous cells

**Table 2:** Primary and secondary endpoints and safety endpoints

	Patient 1	Patient 2	Patient 3
Primary endpoint (month 2)			
VH (reduction) score	0.5+	0.5+ (−1)	0 (−1.5)
Secondary endpoint (month 6)			
BCVA in ETDRS-letters (gain)	82 (+17)	79 (+24)	69 (+11)
CRT in μm (reduction)	265 (−186)	319 (−1)	267 (−83)
Concomitant medication (change)	CSA 2.1 mg/kg bodyweight daily, (+Decortin 0.1 mg/kg bodyweight once daily)	Decortin 0.2 mg/kg bodyweight once daily	MTX 6.25 mg/m^2^/once weekly (−MTX 2.5 mg weekly; − Decortin 0.03 mg/kg bodyweight once daily)
Safety endpoints (month 6)			
IOP	19	11	13
Number of IOP lowering medications during study course (topical or oral)	3 (topical)	2 (topical)	5 (4 topical, 1 oral)
Number of IOP lowering surgeries during study course	0	0	0
CDR (increase)	0.2	0.2	0.2 (+0.1)
Cataract status	clear	clear	Subcapsular cataract grade 2+ (no progression)

*VH* vitreous haze, *BCVA* best corrected visual acuity, *ETDRS- letters* Early Treatment of Diabetic Retinopathy- letters, *CRT* central retinal thickness, *IOP* intraocular pressure, *CDR* cup/ disc- ratio optic nerve head

**Fig. 1:**
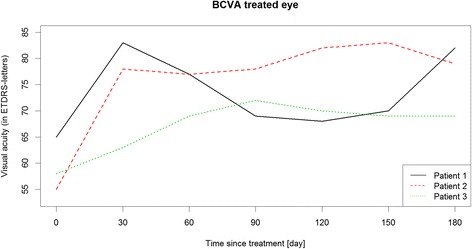
BCVA of the treated eyes during study course

**Fig. 2:**
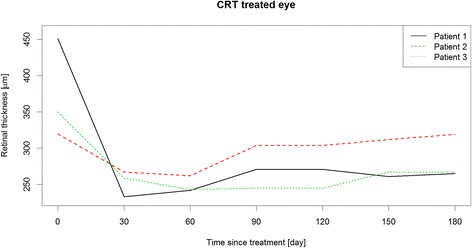
CRT of the treated eyes during study course

**Fig. 3:**
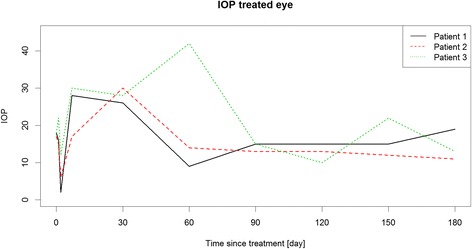
IOP of the treated eyes during study course

### Patient 1

Idiopathic intermediate uveitis was diagnosed at age 16 in this female patient. She had been treated twice with steroid pulse therapy before initiation of oral CSA therapy (2.1 mg/kg body weight daily). CME occurred after 7 months of CSA therapy, thus it was decided to inject DEX implant. After injection, BCVA improved from 65 to 83 letters. CRT was reduced from 451 μm to 233 μm and VH from a score of 0.5+ to 0 one month after injection. Systemic immunosuppressive therapy was reduced to CSA 1.6 mg/kg body weight daily at this time point and further to CSA 1.1 mg/kg body weight daily 2 months after DEX injection. Four months after implantation, an increased VH (2+) was recorded along with decreased BCVA (68 letters). CSA therapy was increased to 2.1 mg/kg body weight daily and a rescue treatment of oral prednisolone 0.1 mg/kg body weight once daily was added. BCVA improved to 82 letters, VH to 0.5+ and CRT was reduced to 265 μm 6 months after injection.

There was a transient hypotony (IOP 2 mmHg) 1 day after DEX injection without evidence of leakage at the sclerotomy site. Topical cycloplegics were prescribed and 7 days after DEX implantation IOP had increased to 28 mmHg. Topical cycloplegics were discontinued and Timolol-Dorzolamide eye drops twice daily and Latanoprost eye drops at bedtime were initiated. Two months after DEX injection IOP was 9 mmHg and IOP lowering eye drops were discontinued at month 5. Six months after DEX implantation, IOP was 19 mmHg without therapy. The cup-to-disc ratio (CDR) of the optic nerve head did not show any progression and the lens remained clear. After study completion intraocular inflammatory activity increased again, but the patient declined further DEX insertion. As a consequence immunosuppressive therapy was augmented consecutively to CSA 6.25 mg/kg body weight daily, Decortin 0.15 mg/kg body weight daily and MTX 10.34 mg/m^2^/orally once weekly. Systemic therapy was then changed to Adalimumab 40 mg subcutaneously every 2 weeks and MTX 10.34 mg/m^2^/orally once weekly and currently intraocular inflammation is well controlled with a VA value of 75 ETDRS letters.

### Patient 2

This 9 year-old male patient had a VH score of 1.5+ units secondary to intermediate uveitis in his left eye. Uveitis initially occurred at age of 6. Sarcoidosis was suspected because of serum IL2- receptor elevation to 969 IU/ml, but no other organ involvement was found during systemic work-up. He had been treated with CSA orally and MTX subcutaneously, but had to discontinue this regimen due to side effects. Upon enrollment, he was prescribed oral Prednisolone at a concentration of 0.2 mg/kg body weight once daily for 3 months without sufficient effect on VH. After DEX injection, VH decreased from 1.5+ units to 0 at the 4 and 6 months time points. BCVA improved from 55 to 83 letters 5 months after DEX injection. Part-time occlusion therapy of the fellow eye was started at month 2. Macular OCT showed diffuse thickening (CRT 320 μm) at baseline but without evidence of CME. CRT decreased to 262 μm at 2 months after DEX injection and then increased to 319 μm at study end. IOP was 17 mmHg prior to DEX injection and 30 mmHg one month after. Timolol-Dorzolamide eye drops were administered twice daily and IOP decreased to 14 mmHg (month 2) and 13 mmHg (month 3), and medication was stopped. At study completion CDR was stable and the lens remained clear. During the following year VA remained stable and VH score increased slightly to 0.5+ units. One year after study completion a relapse occurred, but was effectively controlled with steroid pulse therapy. Currently, no therapy is needed for intraocular inflammation and the patient has a VA of 80 ETDRS letters.

### Patient 3

Primary manifestation of intermediate uveitis occurred at age 15 in this patient. MTX was started after 2 oral steroid pulse therapies. CME and VH persisted during a 6 month period before study inclusion, despite therapy with oral MTX 7.5 mg/m^2^/once weekly and oral Prednisolone 0.03 mg/kg body weight once daily. The patient was treated with DEX implant (use of prednisolone eye drops 5 times daily had not led to increased IOP). After DEX injection, VH score was reduced from 1.5+ units at baseline to 0 units at months 1 to 5. BCVA improved from 58 letters to 72 letters 3 months after DEX implant despite a pre-existing posterior subcapsular cataract. CRT decreased from 350 μm at baseline to a minimum of 243 μm 2 months after DEX injection. IOP increased from 18 mmHg at baseline to 42 mmHg at the 2 months visit. Therapy with Timolol-Dorzolamide eye drops 3 times daily, Latanoprost eye drops once daily and Brimonidine eye drops 3 times daily in combination with oral acetazolamid 250 mg 4 times daily was necessary to lower the IOP to 15 mmHg 3 months after DEX injection. At end of study cataract had not progressed but CDR had increased from 0.1 at baseline to 0.2. After study completion disease activity remained stable with therapy of oral MTX 6.25 mg/m^2^/once weekly. Two and a half years after study completion VA remains stable at 79 ETDRS letters, as do CRT and IOP.

## Discussion

To our knowledge this is the first prospective exploratory case series to evaluate DEX implants in paediatric patients with uveitis. Its major limitation is the small sample size and long term conclusions can not be drawn from these data because patients were only treated once and the follow-up period was only 6 months. However, treatment effect remained stable in patient 2 for 1 year post treatment. To date, treatment effect has also persisted in patient 3 over 2.5 years after study completion applying reduced systemic therapy.

Several reports [30–34] have included more children and adolescents, but all were retrospective data analyses without strict study criteria and therefore with more heterogeneous study groups.

Our results are encouraging with regard to BCVA. The improvement of 11, 17 and 24 ETDRS letters is significant and was associated with a reduction of VH score in patients 2 and 3. During the HURON study, mean change of baseline BCVA in letters was 14 ETDRS letters 8 weeks after DEX treatment and 11 ETDRS letters at week 26 [13]. Additionally, CME improved in patients 1 and 3, who had been treated with DEX implant for this reason. Re-treatment was not necessary in patient 2 and 3, while patient 1 did not wish a re-treatment after study completion.

Bratton et al. [30] retrospectively studied 14 eyes of 11 children (mean age 10.1 years), who received 22 DEX implants for various diagnoses. Two of these 11 children had anterior uveitis and 4 patients were aphakic. Among uveitis specialists aphakia and glaucoma requiring more than 2 medications are the most common contraindications for DEX injections [35]. The authors reported on 4 implants having migrated into the anterior chamber, which is a known possible complication in aphakic patients and was therefore an exclusion criterion in our case series. Mean baseline BCVA improved from 0.9 logMAR to 0.71 logMAR [corresponds to an approximate improvement from 40 to 50 letters using the visual acuity conversion chart of Rosser [36]] 1–3 months after DEX implant. Control or improvement of intraocular inflammation was seen after 17 of 22 DEX injections (12 eyes, 77%). The authors did not analyze VH and CRT, so that a comparison with our findings is not possible.

Another retrospective study of 2 tertiary medical centers enrolled 14 eyes of 10 patients [31]. The study included 2 steroid responders. Other indications for DEX implantation were retinal dystrophy with secondary inflammation, 1 postoperative intraocular inflammation with CME after cataract surgery and 1 case with severe exudative chorioretinal disease presumed to be Vogt-Koyanagi-Harada syndrome. BCVA increased from 0.73 logMAR to 0.53 logMAR [corresponds to an approximate improvement from 50 to 60 letters [36]] 3 months after DEX implantation, accompanied by a decrease in intraocular inflammation (93% of eyes) and a reduction in CRT in all eyes. Recommencing worsening was seen 3–6 months after DEX implantation.

Lei and Lam [32] retrospectively analyzed 4 children (5 eyes) with uveitis (3 eyes), type 1 idiopathic macular teleangiectasia (1 eye) and Coats disease (1 eye), who received a total of 15 DEX implants. BCVA improved from 0.85 ± 0.3 logMAR at baseline to 0.71 ± 0.27 logMAR (mean ± standard deviation) [corresponds to an approximate improvement from 42 to 50 letters [36]] and CRT decreased from 587 ± 185 to 406 ± 135 μm 3 months after treatment. IOP elevation of ≥10 mmHg was noted in 3 eyes and significant lens opacification in 2 eyes.

The largest retrospective study so far, published by Tomkins-Netzer included 22 eyes of 16 children with intermediate or posterior uveitis [33]. BCVA and CRT had improved initially, but returned to baseline at the 6 month visit, although improvement in VH was sustained for longer periods. BCVA increased from 0.55 logMAR at baseline to 0.37 logMAR [corresponds to an approximate improvement from 57 to 67 letters [36]] 1 month after DEX treatment, but decreased again to baseline values at month 6. CRT decreased by 219 μm ± 55 μm (*p* = 0.01) 2 months after DEX insertion and reached baseline values again at the 6 month visit. The percentage of children with a VH of 0 increased from 41% to 88% through DEX implantation and remained stable till month 6. Median time to recurrence was 9 months after the 1st DEX implant and 6 months after the 2nd DEX implant. In contrast to our case series, five patients did not receive systemic imunosuppressive therapy. It is possible that systemic therapy could have further reduced the recurrence rate.

Successful reduction of systemic therapy after DEX treatment was demonstrated in our 3rd patient, while it resulted in increased inflammatory activity in our 1st patient making a rescue treatment necessary. Interestingly, Tomkins-Netzer found a rather low rate of raised IOP (0.21 per eye-year). Of the 10 eyes with known steroid response an increased IOP >21 mmHg was measured in only 4 eyes 2 months following DEX implantation and 1 eye needed revision of the preexisting filtration surgery, whereas we observed an IOP rise in every study patient. This may be due to the low number of patients included.

Dexamethasone seems to have a favourable side effect profile in respect to induction of cataract formation and progression as well as IOP elevation when compared to fluocinolone and triamcinolone [37–39]. This is due to the fact that a binding of steroids to lens and trabecular meshwork depends on lipophilicity. While fluocinolone possesses a higher lipophilicity than triamcinolone, both compounds show a higher lipophilicity than dexamethasone. Furthermore it is well known that steroids induce a trabecular meshwork outflow resistance and several possible mechanisms have been proposed [39]. In addition, the risk for steroid induced glaucoma is higher in children than in adults [40] and it has to be considered that children below the age of 12 have a lower IOP level than adults [41]. Results of our own as well as other studies indicate that DEX implants should be used with caution in paediatric patients with known steroid response, glaucoma or other risk factors for glaucoma. [40, 42].

Patel [43], published his experiences with FA- implants in 4 children (6 eyes) aged 6–13 years. BCVA improved by ≥3 lines (≥ 15 letters) in 3 eyes and intraocular inflammation was controlled in all 6 eyes. Four eyes showed IOP increases ≥30 mmHg and 2 eyes ≥40 mmHg, thus requiring glaucoma surgery.

Another study by Sallam and colleagues [44] reported on “short-term safety and efficacy of intravitreal triamcinolone acetonide for uveitic macular edema in children”, presented as a retrospective case series of 15 children (16 eyes). CME resolved in all eyes and BCVA improved from a mean of 1.0 ± 0.5 logMAR to 0.5 ± 0.3 logMAR (*p* < 0.001) [corresponds to an approximate improvement from 35 to 60 letters [36]]. However, an IOP increase of ≥15 mmHg was seen in 5 eyes and a steroid-induced cataract was recorded in 6 of 11 phakic eyes.

In adults treated with a single DEX implant for noninfectious intermediate or posterior uveitis during the HURON study, < 5% of eyes experienced an IOP of ≥35 mmHg and <10% an IOP of ≥25 mmHg [13]. The MEAD study, where patients with diabetic macular edema (DME) were treated with DEX implants over a time period of 3 years, showed that a steroid induced cataract in adults can not be expected after 1 DEX implant, which was also confirmed in our patients [45]. During the MEAD study most cataract surgeries were performed after 18 to 30 months and repeated DEX implants. The above mentioned studies [30–33] may indicate that development of cataract occurs earlier in paediatric patients than in adults. But in most cases the induced cataract did not yet seem to impact relevantly on VA. In addition it has to be noted that long standing and insufficiently controlled uveitis itself leads to earlier cataract formation and progression in children than in adults.

## Conclusions

The results of our prospective exploratory case series suggest that intravitreal DEX implantation in paediatric patients with idiopathic intermediate uveitis is effective in improving VA and decreasing inflammatory activity. The effect may last much longer than 6 months and reduction of systemic therapy may be possible in some cases. Increased IOP however, is a common complication. Strict in- and exclusion criteria in combination with a low incidence rate of the disease led to a small sample size. Future larger prospective multicenter studies in children and adolescents based on these primary results are necessary to better define indications and contraindications in this age group.

## Abbreviations

AE: Adverse event
AMG: German law for pharmaceutical products
BCVA: Best corrected visual acuity
BfArM: German Institute for pharmaceutical products and medical devices
CDR: Cup-to-disc ratio
CME: Cystoid macular edema
CRT: Central retinal thickness
CSA: Ciclosporine A
DEX: Dexamethasone
EMA: European Medicines Agency
ETDRS: Early treatment of diabetic retinopathy charts
FA: Fluocinolone acetonide
GCP: Good clinical practice
IOP: Intraocular pressure
JIA: Juvenile idiopathic arthritis
LE: Left eye
LOCS: Lens opacity classification system
MTX: Methotrexate
RA: Right eye
SAE: Serious adverse event
SD-OCT: Spectral domain optical coherence tomography
VC: Vitreous cells
VH: Vitreous haze
